# Shift in tree species changes the belowground biota of boreal forests

**DOI:** 10.1111/nph.18109

**Published:** 2022-04-10

**Authors:** Sunil Mundra, Håvard Kauserud, Tonje Økland, Jørn‐Frode Nordbakken, Yngvild Ransedokken, O. Janne Kjønaas

**Affiliations:** ^1^ Section for Genetics and Evolutionary Biology (EvoGene) Department of Biosciences University of Oslo PO Box 1066 Blindern Oslo NO‐0316 Norway; ^2^ Department of Biology College of Science United Arab Emirates University PO Box 15551 Al‐Ain, Abu‐Dhabi United Arab Emirates; ^3^ Norwegian Institute of Bioeconomy Research PO Box 115 Ås NO‐1431 Norway; ^4^ Faculty of Environmental Sciences and Natural Resource Management Norwegian University of Life Sciences PO Box 5003 Ås NO‐1432 Norway

**Keywords:** boreal forest, carbon and nitrogen stock, downy birch (*Betula pubescens*), ectomycorrhiza, fungal guild, Norway spruce (*Picea abies*), tree species effects

## Abstract

- The replacement of native birch with Norway spruce has been initiated in Norway to increase long‐term carbon storage in forests. However, there is limited knowledge on the impacts that aboveground changes will have on the belowground microbiota.
- We examined which effects a tree species shift from birch to spruce stands has on belowground microbial communities, soil fungal biomass and relationships with vegetation biomass and soil organic carbon (SOC).
- Replacement of birch with spruce negatively influenced soil bacterial and fungal richness and strongly altered microbial community composition in the forest floor layer, most strikingly for fungi. Tree species‐mediated variation in soil properties was a major factor explaining variation in bacterial communities. For fungi, both soil chemistry and understorey vegetation were important community structuring factors, particularly for ectomycorrhizal fungi. The relative abundance of ectomycorrhizal fungi and the ectomycorrhizal : saprotrophic fungal ratio were higher in spruce compared to birch stands, particularly in the deeper mineral soil layers, and vice versa for saprotrophs.
- The positive relationship between ergosterol (fungal biomass) and SOC stock in the forest floor layer suggests higher carbon sequestration potential in spruce forest soil, alternatively, that the larger carbon stock leads to an increase in soil fungal biomass.

## Introduction

Global climate change is a major threat to the functioning of all ecosystems, including forests, which cover 30% of the Earth’s terrestrial surface (Keenan *et al*., 2015). Historically, plantations of nonnative tree species have been established worldwide to enhance timber production, and, more recently, to mitigate climate change (Bastin *et al*., 2019). Deciduous birch (*Betula pubescens* Ehrh.) and coniferous Norway spruce (*Picea abies* (L.) Karst; hereafter spruce) are dominant forest trees in central Europe as well as in boreal forests, but their distribution and abundance vary between regions. Change in tree species from native birch to faster‐growing spruce has been proposed in Norway to increase annual CO_2_ uptake and long‐term carbon (C) storage in forests (Norwegian Ministry of Environment, 2012). In western Norway, the consequences of these changes include shifts in the structure and biomass of understorey vegetation, changes in soil C stocks and chemistry, and a substantial increase in the total ecosystem C in spruce (357 ± 22 Mg ha^−1^) compared to birch stands (208 ± 7 Mg ha^−1^) (Kjønaas *et al*., 2021).

Understanding the linkages between aboveground and belowground biotic pools is important for advancing knowledge of forest ecosystems and processes (Wardle *et al*., 2004). In forests, plant–microbial interactions are often mediated by the dominant tree species (Tedersoo *et al*., 2015; Urbanová *et al*., 2015), which in turn directly or indirectly affect soil microbial composition (Prescott & Grayston, 2013; Šnajdr *et al*., 2013) by, for example, affecting understorey vegetation (Ma, 2019), changing soil physico‐chemical properties (Ayres *et al*., 2009; Danielsen *et al*., 2021), producing different types of aboveground and belowground litter (Wardle *et al*., 2004), rhizodepositing easily degradable C compounds (Jones *et al*., 2004) and secreting root exudates (Bais *et al*., 2006). Microorganisms further contribute to total soil respiration (Högberg *et al*., 2001, 2010) and to C stocks in living biomass and soil (Clemmensen *et al*., 2013). Several ectomycorrhizal fungi are known to form different exploration types (Agerer, 2001, 2006). The short‐distance, contact and smooth exploration types have a greater ability to access labile nutrient pools at a lower cost to the host plant compared with fungi possessing long‐distance exploration types, which are better adapted to complex organic resources (Lilleskov *et al*., 2011). Thus, different exploration types help ectomycorrhizal fungi to acquire nutrients from organic and mineral layers, potentially affecting nutrient dynamics through the hyphal network connected to plant roots. Considering the crucial role of the microbiota in global C dynamics, nutrient cycling and soil organic matter (SOM) build‐up in forest ecosystems, it is imperative to understand the effect of shifts in dominant tree species on soil microbial communities (Wardle *et al*., 2011).

Changes in soil characteristics, such as soil chemistry, soil acidity, humus composition, soil organic C (SOC) and nitrogen (N) stocks, may be related to differences in litter inputs (Kriiska *et al*., 2019), as well as decomposition rates in birch and spruce stands (Hansson *et al*., 2011; Mueller *et al*., 2012; Olsson *et al*., 2012), which in turn may be linked to differences in soil microbial species composition. Spruce typically has a shallow subsurface rooting pattern, whereas birch is more deeply rooted (Puhe, 2003; Hansson *et al*., 2013a). This may affect soil microbial community composition through root litter input and root exudates from the upper organic forest floor, as well as the mineral soil (Moll *et al*., 2015). Strong impacts of tree species change have been found on tree biomass, decomposition rates and potentially also forest productivity (Hansson *et al*., 2013a; Kjønaas *et al*., 2021), but the extent to which tree species affect quantitative and qualitative traits of belowground microbial communities remain unknown (Waller *et al*., 2020).

Fungi typically have strong links to plants through mycorrhizal symbioses, which enhance plant access to soil nutrients. Small‐sized, fast‐growing bacteria, however, inhabit soil niches of broader nutritional ranges (Boer *et al*., 2005) and show a preference towards root exudates (Zhalnina *et al*., 2018). Fungi are primarily responsible for controlling N‐poor plant litter decomposition, whereas N‐rich microbial biomass is thought to be decomposed largely by bacteria (Brabcová *et al*., 2018; López‐Mondéjar *et al*., 2018). The filamentous nature of fungi, coupled with their ability to tolerate low pH and to secrete extracellular enzymes (including ligninolytic peroxidases), thus facilitating co‐metabolic degradation of polymer and lignin, makes fungi well suited to the decomposition of SOM in forest soils (Lindahl *et al*., 2002; Baldrian & Valášková, 2008; Bödeker *et al*., 2014; Větrovský *et al*., 2014; Gallardo *et al*., 2021). However, evidence also supports the participation of free‐living bacteria in litter transformations (Baldrian *et al*., 2012; Žifčáková *et al*., 2017) and the breakdown of cellulose, hemicellulose and other polysaccharides (Štursová *et al*., 2012; López‐Mondéjar *et al*., 2016). Additionally, due to their ability to produce extracellular enzymes such as lytic chitin monooxygenase and chitinase, bacteria may feed on fungal biomass (Starke *et al*., 2020). Although the decomposition efficiency of bacteria is considered to be much lower than that of fungi (Brown & Chang, 2014), they share similar niches in soil with fungi. They may, however, not respond in a similar way to changes in aboveground biota, due to their looser relationships with tree species.

A wide range of edaphic factors such as pH, nutrient availability, quantity and quality of SOC, including root‐derived C, vary with soil depth. Accordingly, a corresponding shift in microbial communities with depth is expected. Bacteria and fungi often respond to pH, whereas variability in soil C and N concentrations strongly affects fungal community composition (Lauber *et al*., 2008; Bahram *et al*., 2018). Saprotrophic fungi largely colonize the litter of the upper forest floor layer as well as more decomposed plant material (Voříšková *et al*., 2014). With increasing soil depth, the abundance of saprotrophic fungi declines and ectomycorrhizal fungi dominate the lower humus and mineral soil layers due to changes in substrate quality, C : N ratio and increasing competitive pressure from root‐associated fungi (Lindahl *et al*., 2007; Edwards & Zak, 2010). Fungal hyphae originating from host roots in the forest floor layer may extend further down into the mineral soil (Koide *et al*., 2005), and exert impacts on local bacterial communities by providing a specific bacterial niche, known as the mycorrhizosphere.

Plant litter is the major contributor to SOM accumulation in the forest floor layers, with litter chemistry being highly variable between tree and understorey plant species. Therefore, an effect of tree species on soil microbial communities in the forest floor layer can be expected (Prescott & Grayston, 2013; Augusto *et al*., 2015). Furthermore, compared with mineral soil layers, higher microbial biomass has been reported in forest floor layers in stands of birch (Mundra *et al*., 2021) and other tree species (Baldrian *et al*., 2013). In contrast, effects of different tree species in the mineral soil has been suggested to be mainly driven by direct microbial interactions with roots (Whalen *et al*., 2021) or by microorganisms utilizing tree species‐specific root exudates (Bais *et al*., 2006), and may further be governed by variation in local soil chemistry (Tedersoo *et al*., 2015). Soil processes, soil properties and microbial communities are depth‐dependent, and for a more complete understanding, studies on soil characteristics beyond the surface layer are needed (Yost & Hartemink, 2020), including comparisons of microbial communities at different soil depths.

A recent companion study to the research reported here assessed the effects of a shift from unmanaged birch to planted spruce on ecosystem C balance (Kjønaas *et al*., 2021). It showed lower vascular plant cover and understorey vegetation C and N stocks, higher forest floor C stocks and changes in exchangeable nutrients, acidity, and base saturation under spruce (Kjønaas *et al*., 2021). In the present study, we investigated the impacts of the shift from birch to spruce on belowground microbial communities along a soil depth gradient, with emphasis on their relationship with ground vegetation and soil characteristics, as well as SOC and N stocks. We hypothesized that: (Hypothesis 1) fungal communities would show greater response to change in tree species compared with bacteria, since many fungi are tightly associated with a host tree, (Hypothesis 2) the impact of tree species change on microbial communities would be stronger in the upper organic layer compared with the mineral soil, and (Hypothesis 3) microbial richness will be negatively impacted by the decrease in understorey vegetation biomass and vascular plant cover in spruce forests.

## Materials and Methods

### Site description and sampling

Our study was conducted in native, naturally occurring birch stands (*B. pubescens*) and adjacent mature stands of spruce (*Picea abies*) that had been planted on previous birch forest. Four study locations were selected in the counties Vestland and Møre and Romsdal in western Norway (Supporting Information Fig. S1a). Historically, the birch stands have been subjected to rough grazing and selective harvesting. The spruce stands were relatively dense compared with the birch, with a total age of *c*. 45–60 yr (Kjønaas *et al*., 2021). The understorey vegetation in all birch stands consisted of a mix of dwarf shrubs (mainly *Vaccinium myrtillus* L.), herbs, ferns, graminoids and bryophytes. The understorey vegetation in the spruce stands was dominated by bryophytes that varied in density and distribution. Detailed site descriptions, sampling design, the impact of tree species change on tree biomass, understorey vegetation cover, soil chemistry and ecosystem SOC and N stocks are reported in the Methods S1 and by Kjønaas *et al*. (2021).

Within each stand, three 144 m^2^ plots were established by selecting homogeneous soil and stand conditions, and 20 soil cores (Ø = 2.6 cm) were sampled in a grid design. Each soil core was divided into four layers: the forest floor (horizons of forest litter at various stages of decomposition; hereafter LFH layer) and three mineral soil layers (0–5 cm (M1); 5–15 cm (M2) and 15–30 cm (M3)). The LFH horizons were sampled as one layer due to their highly variable thickness and composition, ranging from litter with no ground flora to a continuous and thick cover of mosses, a highly variable degree of litter being mixed into the floor (F) horizon, as well as varying presence of litter (L), F and humus (H) horizons within and between plots and locations. The 20 cores from each plot were pooled into one composite sample per layer, resulting in a total of 96 samples (4 locations × 2 tree species × 3 plots × 4 depths). Samples for DNA and ergosterol analyses were stored at −20°C immediately after collection, whereas samples for chemistry analysis were kept cool during transport and frozen immediately after returning them to the laboratory. Prior to the DNA and ergosterol analyses, the soil was homogenized by passing it through a 2 mm sterilized sieve, followed by freeze‐drying and pulverizing using a FastPrep instrument (MP Biomedicals, Illkirch‐Graffenstaden, France).

### Ergosterol and soil chemistry analyses

We measured concentrations of ergosterol (as a proxy for fungal biomass) in freeze‐dried and finely ground soil fractions, following the protocol of Ransedokken *et al*. (2019). Methods on soil chemistry (exchangeable elements, pH, organic matter and C and N concentrations), soil SOC and N stocks, understory vegetation cover and biomass and aboveground C and N stocks are given in Kjønaas *et al*. (2021).

### DNA extraction and Illumina sequencing

DNA was extracted from 1 ***g*** freeze‐dried soil and purified using E.Z.N.A soil DNA kit (Omega Biotek, Norcross, GA, USA). Following the PCR reaction setup and library preparation methods of Mundra *et al*. (2021), we amplified the 16S rRNA region of bacteria using the primers 515F (5′‐GTGCCAGCMGCCGCGGTAA‐3′) and 806R (5′‐GGACTACHVHHHTWTCTAAT‐3′) (Caporaso *et al*., 2011), and the ITS2 region of fungi using the primers fITS7a (5′‐GTGARTCATCGARTCTTTG‐3′) and ITS4 (5′‐TCCTCCGCTTATTGATATGC‐3′) (White *et al*., 1990; Ihrmark *et al*., 2012). Two negative control and six technical replicates (samples sequenced twice with different tags) were included in both bacterial and fungal sequencing runs, and amplicons were sequenced using the Illumina Miseq (paired‐end 2 × 300 bp) platform by StarSEQ GmbH, Mainz, Germany.

### Bioinformatic analyses

The 16S and ITS2 sequence datasets were analysed using a similar bioinformatic pipeline to that described by Mundra *et al*. (2021). We performed error correction with BayesHammer (Nikolenko *et al*., 2013), reads merging with Pear v.0.9.10 (Zhang *et al*., 2014), quality filtering using Fastx‐Toolkit v.0.0.14 and Vsearch v.2.4.3 (Rognes *et al*., 2016) and demultiplexing using Sdm v.1.41 program (Hildebrand *et al*., 2014). We used ITSx v.1.0.11 to extract the ITS2 region from the fungal dataset (Nilsson *et al*., 2010). After the filtering steps, 5.8 million bacterial reads (raw reads 7.5 million), and 17.1 million fungal reads (raw reads 18.6 million) remained in the datasets. Vsearch was used for dereplication, global singletons removal and sequence clustering at the 97% similarity threshold. Chimera analysis was performed on representative sequences using the ‐‐*uchime_denovo* algorithm (Edgar *et al*., 2011). Operational taxonomic units (OTUs) with < 10 reads and chimera and global singletons were removed, which resulted in 2717 and 6020 OTUs in the 16S and ITS2 datasets, respectively.

Taxonomic assignments of bacteria and fungi were performed using Greengene v.13.8 (DeSantis *et al*., 2006; McDonald *et al*., 2012) and unite v.6 (Kõljalg *et al*., 2013), using Blast, respectively. The OTU table from the ITS2 dataset was passed through FUNGuild (Nguyen *et al*., 2015) to assign fungi into functional guilds (ectomycorrhizal fungi and saprotrophic fungi) with a confidence ranking of highly probable and probable. Furthermore, ectomycorrhizal fungal genera were annotated with their exploration types (Agerer, 2001, 2006) and classified into two categories, namely, contact, short, medium distance smooth (C.S.MDS) and long and medium distance fringe and mat (L.MDF.MDM).

In the ITS dataset, we excluded (i) OTUs with no blast hits (1539), (ii) OTUs having < 80% coverage and identity to a fungal reference (318), (iii) OTUs of plants (15), and (iv) a single protist taxon, resulting in 4147 OTUs in the final dataset. From the 16S dataset we removed OTUs identified as Archaea (65) and one OTU (matching to *Micrococcus*) present in a control sample, which resulted in a final dataset of 2651 OTUs. Prior to the richness analyses, we normalized both datasets to a similar number of reads per samples (16S = 6154; and ITS2 = 50 938). Overall sequence data characteristics, microbial community composition and replicated samples results consistency (Fig. S2a,b) are presented as Notes S1.

### Statistical analyses

To reduce variance heterogeneity, OTU data were arcsine‐transformed, whereas soil and understorey vegetation variables were transformed to zero skewness (normal distribution) and standardized on a 0–1 scale. To account for covariance, significantly correlating variables were grouped together based on principal component analysis (PCA) using the *stats* package in R (R Core Development Team, 2020). PCA scores for biomass and cover of understorey vegetation species groups (namely, woody plants (including dwarf shrubs and small trees), herbs, ferns and graminoids, and bryophytes) were strongly correlated (*R* ^2^ = 0.984, *P* > 0.001), and hence only results for plant biomass are reported (Fig. S3a). The first axis score generated from a PCA on biomass data was termed ‘understorey vegetation biomass index’ (biomass PC1), representing the biomass of dwarf shrubs and small trees, and that of graminoids, herbs and pteridophytes in the birch stands (Kjønaas *et al*., 2021). The first axis of the PCA performed on soil properties was termed a ‘depth related soil variability index’ (soil PC1) and represents the decline in SOC, N and other nutrients at greater depth in the soil profile (Fig. S3b,c). The second axis of the PCA was termed ‘tree species related soil variability index’ (soil PC2) and reflects tree species‐related variation in soil chemistry, specifically the higher concentrations of N in the LFH layer and those of calcium (Ca) and magnesium (Mg) in the M1 layer in birch stands compared with spruce, as well as higher exchangeable acidity, C : N ratio and concentrations of C, potassium (K) and sodium (Na), in the LFH layer of spruce stands compared with birch (Kjønaas *et al*., 2021).

To examine the effects of the change from birch to spruce on ergosterol concentration, bacterial and fungal richness, and the relative abundances of fungal functional guilds (ectomycorrhiza, saprotrophs and their ratio) at the four soil depths, we employed ANOVA followed by Tukey’s HSD *post hoc* tests using the package agricolae (Mendiburu, 2020). To investigate the effects of soil PC1, soil PC2 and biomass PC1 on microbial parameters and the relative abundances of dominant bacterial phyla (namely, Proteobacteria, Acidobacteria, Firmicutes, Actinobacteria, Planctomycetes, Verrucomicrobia, Chloroflexi and Bacteroidetes), linear mixed effect (LME) models were fitted using the package nlme (Pinheiro *et al*., 2014). Similar LME models were also run to assess the response of SOC and N stocks against earlier‐mentioned microbial properties. The LME models were run assuming Poisson distribution for count data variables (richness), and normal distribution for the relative abundances of bacterial phyla, fungal functional guilds, and SOC and N stocks. Sampling location and plots nested in a location were included as random factors in all models.

To perform multivariate analyses for bacteria and fungi (overall, ectomycorrhizal fungi and saprotrophic fungi), we calculated Bray–Curtis distances. We used the *varpart* function of the package vegan (Oksanen *et al*., 2013) to partition the variation (adjusted *R* ^2^) of bacterial and fungal community dissimilarity, by teasing apart variation related purely to tree species, soil depth, soil chemistry and understorey vegetation biomass variables. Variation partitioning was based on redundancy analysis (RDA), where Euclidean distance matrices of soil chemistry and plant biomass were used. Mantel tests from the package vegan were used to investigate the effects of soil chemistry and understorey biomass distances on bacterial and fungal community composition. The importance of tree species, soil depth and their interactions in explaining bacterial and fungal community composition were assessed by permutational analysis of variance (PERMANOVA) using the Adonis function of the package vegan (9999 permutations for pseudo‐*F* statistics). PERMANOVA analyses were performed using a forward selection procedure in which single factor models were tested first, with significant factors being included in the final model. To avoid multicollinearity, we also repeated PERMANOVA analyses after excluding categorical variables (soil depth and tree species) and tested only vector variables (soil PC1, soil PC2, biomass PC1 and their interactions) using PERMANOVA analyses. The community datasets were also subjected to Global Nonmetric Multi‐Dimensional Scaling (NMDS) ordination analyses with the following settings: distance measure = Bray–Curtis distance; dimensions = 2; initial configurations = 100; maximum iterations = 1000; minimum stress improvement in each iteration = 10^–5^. The *envfit* function was used to investigate the significance of the explanatory variables (regression with NMDS1 and NMDS2). To test the effects of tree species on both bacterial and fungal communities for each of the four soil depths separately, we conducted additional PERMANOVA analyses using the similar settings.

## Results

### Correlations between ergosterol, fungal guilds, SOC, N stocks and biotic and abiotic factors

**Fig. 1:**
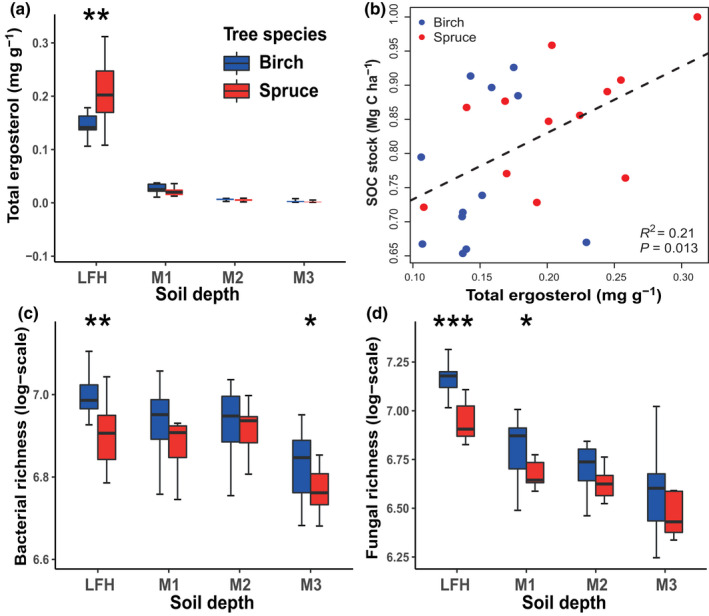
Effects of the change from birch to Norway spruce on belowground microbial properties. Tree species (birch vs spruce) effect on (a) total ergosterol concentration (mg g^−1^) (c) bacterial richness and (d) fungal richness at different soil depths (forest floor (LFH) and three mineral soil layers: 0–5 cm (M1), 5–15 cm (M2), and 15–30 cm (M3)), was tested using ANOVA. (b) A linear regression analysis was performed to investigate the relationship between total ergosterol and soil organic carbon (SOC; Mg C ha^−1^) stock in the LFH (forest floor) layer of birch and Norway spruce stands. Total ergosterol and SOC data were transformed to zero skewness, i.e. normal distribution and scaled on a 0–1 level prior to plotting. The centerline in box plots (a, c, d) represent medians and box limits indicate the 25^th^ and 75^th^ percentiles quartile. The line in plot (b) represents a fitted linear regression. Asterisks indicate significant differences between the two tree species (Significance levels: *, *P* < 0.05; **, *P* < 0.01; ***, *P* < 0.001).

We found a significant effect of tree species change on ergosterol concentration in the LFH layers (Fig. 1a; Table S1), as well as a positive correlation (*R* ^2^ = 0.21, *P* = 0.013) between ergosterol concentration and SOC stocks (Fig. 1b; Table S1), with both variables being higher in the LFH layer of spruce compared with birch stands. The ergosterol concentration declined significantly from the LFH layer to the mineral soil (*F*_3,92_ = 225.6, *P* < 0.001), and was strongly correlated with the depth related soil variability index (soil PC1) (Table 1). There was, however, no significant relationship between ergosterol concentration and the tree species related soil variability index (soil PC2), or between ergosterol concentration and the understorey vegetation biomass index (biomass PC1). The relative abundances of fungal functional groups displayed no relationships with either the SOC or the N stocks in any of the soil layers (Table S1).

**Table 1:** Linear mixed effect (LME) models analysing the relationship between soil and understorey biomass related principal component analysis (PCA) axes and microbial properties.

	Soil PC1		Soil PC2		Biomass PC1	
	*t*‐value	*P*‐value	*t*‐value	*P*‐value	*t*‐value	*P*‐value
*Fungi*						
OTUs richness	−10.76	**< 0.001**	1.24	0.217	−2.54	**0.013**
Ectomycorrhizal fungi	5.51	**< 0.001**	−3.08	**0.003**	5.38	**< 0.001**
Saprotroph	−5.05	**< 0.001**	1.44	0.153	−1.50	0.137
Ectomycorrhizal : saprotrophic fungi ratio	3.54	**< 0.001**	−3.42	**< 0.001**	4.46	**< 0.001**
Ergosterol	−15.41	**< 0.001**	0.05	0.962	0.82	0.415
*Bacteria*						
OTUs richness	−4.45	**< 0.001**	1.75	0.083	−2.98	**0.004**
Proteobacteria	−8.67	**< 0.001**	2.46	**0.016**	−0.70	0.488
Acidobacteria	9.36	**< 0.001**	0.36	0.718	0.54	0.592
Firmicutes	6.75	**< 0.001**	−4.04	**< 0.001**	0.63	0.532
Actinobacteria	−12.57	**< 0.001**	5.94	**< 0.001**	−1.23	0.223
Planctomycetes	−4.57	**< 0.001**	6.07	**< 0.001**	−1.02	0.311
Verrucomicrobia	−10.79	**< 0.001**	1.34	0.183	0.91	0.366
Chloroflexi	11.31	**< 0.001**	−4.54	**< 0.001**	0.08	0.935
Bacteroidetes	−18.64	**< 0.001**	1.50	0.136	−0.67	0.503

OUTs, operational taxonomic units.

The response of different microbial properties (amount of ergosterol, bacterial and fungal richness as well as relative abundance of fungal functional guilds and proportions of dominating bacterial phyla) was examined against fixed effect variables: soil PCA axes (soil PC1: depth related soil variability index; soil PC2: tree species related soil variability index and understorey PCA axis (biomass PC1: understorey vegetation biomass index), using mixed models, with sampling locations as a random factor. Soil PC1 and soil PC2 indices are ordination score of the first and second axes of the PCA carried out for soil chemical properties. Biomass PC1 is the score of first axis of the PCA conducted for the biomass of understorey vegetation. Numbers in bold represent significant (*P* < 0.05).

### Effects of tree species and soil depth on bacterial and fungal richness

We found that OTU richness was significantly lower in spruce compared with birch stands for both bacteria (birch: 1018 ± 102; spruce: 960 ± 85; *F*_1,94_ = 8.09, *P* = 0.005) and fungi (birch: 947 ± 259; spruce: 818 ± 173; *F*_1,94_ = 6.73, *P* = 0.011). Bacterial and fungal OTU richness were significantly higher in the LFH layer of the birch compared with spruce stands, and declined significantly with soil depth (Fig. 1c,d). This was reflected in the mixed model analyses, which displayed a negative relationship between OTU richness and soil PC1 (Table 1). However, a negative relationship was found between bacterial and fungal OTUs richness and understorey biomass PC1 (Fig. S3a). The richness of Actinobacteria was higher in the LFH layer of birch stands, relative to mineral soil layers, whereas Acidobacteria showed the opposite pattern (Table S2). Among the fungi, the richness of the phyla Ascomycota, Glomeromycota and Rozellomycota were higher in birch compared with spruce stands, while the richness of Basidiomycota was higher in spruce than in birch forests.

### Effects of tree species on bacterial and fungal communities

Mantel tests revealed that bacterial community composition correlated more strongly with soil chemistry (*r* = 0.74, *P* < 0.001) than with understorey vegetation biomass (*r* = 0.04, *P* = 0.024). Fungal community composition, however, strongly correlated with both soil properties (*r* = 0.47, *P* < 0.001) and understorey vegetation biomass (*r* = 0.46, *P* < 0.001). Variation partitioning analyses further showed that depth alone accounted for 17% and 4% of the total variation in bacterial and fungal communities, respectively (Fig. S4a,b). The shared effect of tree species and understorey vegetation biomass was stronger for fungi (12%) than for bacteria (1%).

**Fig. 2:**
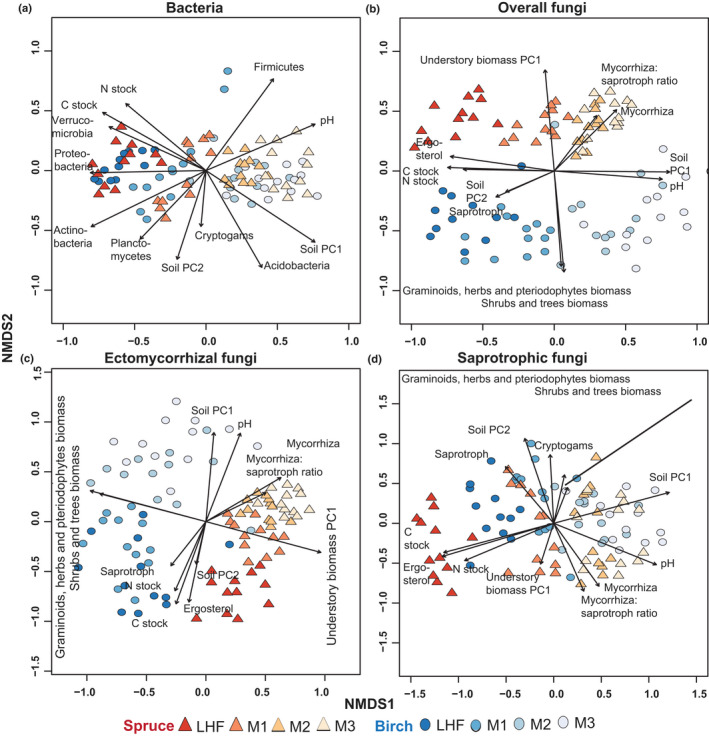
Global nonmetric multidimensional scaling (NMDS) ordination analysis for bacterial and fungal communities. Samples were collected from two forest types dominated by birch and Norway spruce along soil depth gradients (forest floor (LFH) and three mineral soil layers: 0–5 cm (M1), 5–15 cm (M2), and 15–30 cm (M3)) from western Norway. The sample (*n* = 96) based ordination plots for (a) bacteria, (b) overall fungi, (c) ectomycorrhizal fungi and (d) saprotrophic fungi were generated from total operational taxonomic units (OTUs) compositions. The different shapes and colour used in plots indicates forest types (birch, blue; Norway spruce, red) and colour gradient represents depth profile. Arrows point in the direction of maximum increase of individual vector variables and had significant effects (*P* < 0.05) on the ordination configuration. Soil PC1 (depth related soil variability index) and soil PC2 (tree species related soil variability index) are ordination score of the first and second axes of the principal component analysis (PCA) carried out for soil chemical properties. Biomass PC1 (understorey vegetation biomass index) is the score of first axis of PCA conducted for the biomass of understorey vegetation. Carbon (C) stock, soil organic C (SOC) stock; nitrogen (N) stock, total N stock in the soil.

Both multivariate methods showed that the bacterial community was strongly structured with soil depth (*R* ^2^ = 0.51), whereas tree species had limited significance (*R* ^2^ = 0.02; Fig. 2a; Table 2). Further, the tree species × soil depth interaction had no impact on bacterial communities (Table 2). After excluding direct effects of soil depth and tree species from the analyses, we found a strong correlation with soil PC1 (*R* ^2^ = 0.40) and soil PC2 (*R* ^2^ = 0.13) (Table 2). At the phylum level, the relative abundance of Firmicutes was higher in spruce stands, whereas Actinobacteria and Planctomycetes tended to be more frequent in birch stands (Table S3). The relative abundances of these three phyla were significantly associated with soil PC2 (Table 1).

**Table 2:** PERMANOVA analysis showing effects of different biotic and abiotic factors on microbial species community composition structure.

Variables	*F* model	*R* ^2^ value	*P*‐value	Variables	*F* model	*R* ^2^ value	*P*‐value
Excluding PCA axes scores				Excluding tree species and depth			
**Bacteria**							
Tree species	3.73	0.02	**0.012**	Biomass PC1	3.81	0.02	**0.014**
Soil depth	33.22	0.51	**< 0.001**	Soil PC1	81.02	0.40	**< 0.001**
Tree species × soil depth	1.24	0.02	0.183	Soil PC2	26.62	0.13	**< 0.001**
Residuals	0.45			Biomass PC1 × soil PC1	2.26	0.01	0.72
				Residuals	0.44		
**Fungi**							
Tree species	18.18	0.13	**< 0.001**	Biomass PC1	18.17	0.13	**< 0.001**
Soil depth	6.99	0.15	**< 0.001**	Soil PC1	16.23	0.11	**< 0.001**
Tree species × soil depth	2.94	0.06	**< 0.001**	Soil PC2	10.26	0.07	**< 0.001**
Residuals	0.65			Biomass PC1 × soil PC1	6.65	0.04	**< 0.001**
				Residuals	0.64		
**Ectomycorrhizal fungi**							
Tree species	19.59	0.15	**< 0.001**	Biomass PC1	19.69	0.14	**< 0.001**
Soil depth	5.58	0.13	**< 0.001**	Soil PC1	13.54	0.10	**< 0.001**
Tree species × soil depth	2.67	0.06	**< 0.001**	Soil PC2	9.16	0.07	**< 0.001**
Residuals	0.66			Biomass PC1 × soil PC1	5.89	0.04	**< 0.001**
				Residuals	0.65		
**Saprotrophic fungi**							
Tree species	8.95	0.07	**< 0.001**	Biomass PC1	8.86	0.06	**< 0.001**
Soil depth	10.80	0.24	**< 0.001**	Soil PC1	22.77	0.16	**< 0.001**
Tree species × soil depth	2.74	0.06	**< 0.001**	Soil PC2	13.85	0.10	**< 0.001**
Residuals	0.64			Biomass PC1 × soil PC1	4.72	0.03	**< 0.001**
				Residuals	0.64		

Effects of tree species (birch vs spruce), different soil depth (forest floor (LFH) and three mineral soil layers: 0–5 cm (M1), 5–15 cm (M2), and 15–30 cm (M3)) and their interaction were tested on bacterial, fungal, ectomycorrhizal fungi and saprotrophic fungi communities. When analyzing effect of *understorey vegetation biomass index* (biomass PC1), *depth related soil variability index* (soil PC1), and *tree species related soil variability index* (soil PC2), tree species and soil depth, which were corelated with the PCA axes, were excluded from the models. *P*‐values were obtained using 9999 permutations and bold indicates statistical significance (*P* < 0.05).

**Fig. 3:**
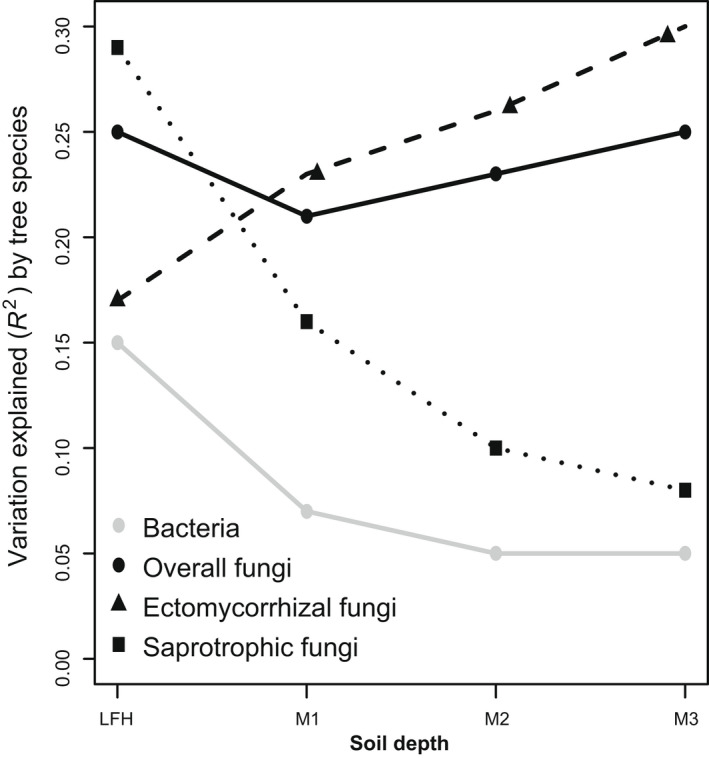
Effect of the change from birch to Norway spruce on microbial communities at different soil depths. Adonis (PERMANOVA) analysis was used to calculated proportion of variation explained (*R* ^2^) by tree species for bacteria, overall fungi, ectomycorrhizal fungi and saprotrophic fungi communities at different soil depth (forest floor (LFH) and three mineral soil layers: 0–5 cm (M1), 5–15 cm (M2), and 15–30 cm (M3)). All the tested model explained variation at the significance level of *P* < 0.05. The solid line with grey and black colour represents explained variation in bacterial and overall fungal communities, respectively. Dash line with triangle and square shape represents explained variation in ectomycorrhizal and saprotrophic fungal communities, respectively.

Contrary to bacteria, the composition of the fungal community was correlated with both tree species (*R* ^2^ = 0.13) and soil depth (*R* ^2^ = 0.15) (Fig. 2b; Table 2). Further, the PERMANOVA analyses conducted for each separate soil layer showed that tree species had a stronger effect on fungal communities in all soil layers than it did on bacteria (Fig. 3). After excluding direct effects of soil depth and tree species from the analyses, we found that soil PC1, soil PC2 and biomass PC1 were also significant determinants of fungal community composition (Table 2).

### Effects of tree species on fungal guilds and composition

**Fig. 4:**
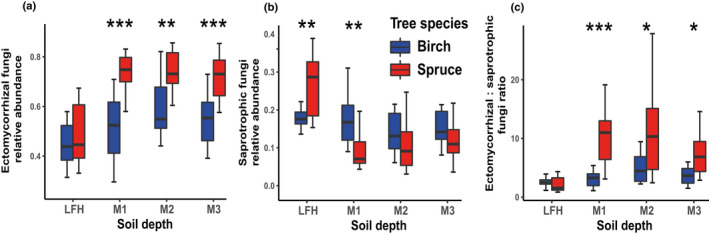
Effects of the change from birch to Norway spruce on belowground fungal guilds proportions. Tree species (birch vs spruce) effect on the relative abundance of (a) ectomycorrhizal fungi, (b) saprotrophic fungi and (c) ectomycorrhizal : saprotrophic fungi ratio at different soil depths (forest floor (LFH) and three mineral soil layers: 0–5 cm (M1), 5–15 cm (M2), and 15–30 cm (M3)) was tested using ANOVA. The centerline in box plots (a–c) represent medians and box limits indicate the 25^th^ and 75^th^ percentiles quartile. Asterisks in the different panels indicates significant differences between the two tree species (Significance levels: *, *P* < 0.05; **, *P* < 0.01; ***, *P* < 0.001).

Both ectomycorrhizal and saprotrophic fungal communities were structured according to tree species, but the tree species effect was more pronounced for the former guild (Fig. 2c,d; Table 2). The analysis of fungal functional guilds showed that the abundance of ectomycorrhizal fungi was proportionally higher in spruce stands (*F*_1,118_ = 26.23, *P* < 0.001). The difference was highly significant in all three mineral layers, but not in the LFH layer (Fig. 4a). The relative abundance of saprotrophic fungi was similar in both forest types (*F*_1,94_ = 2.01, *P* = 0.159); however, it was higher in the LFH layer of the spruce stands, and higher in the M1 layer of the birch stands (Fig. 4b). Thus, the saprotrophic community response to tree species was stronger in the LFH layer than it was in the mineral soil layers, whereas ectomycorrhizal fungal communities displayed the opposite response (Fig. 3). The saprotrophic fungal community showed a weaker relationship with biomass PC1 compared with soil PC1 and soil PC2, whereas ectomycorrhizal fungi showed opposite patterns (Table 2). The ectomycorrhizal : saprotrophic fungal ratio was significantly higher in the spruce stands (*F*_1,94_ = 16.33, *P* < 0.001), with dominance of ectomycorrhizal fungi over saprotrophic fungi in all three mineral layers (Fig. 4c). The abundance of ectomycorrhizal fungi and the ectomycorrhizal : saprotrophic fungal ratio were correlated with soil PC1, soil PC2 and biomass PC1, indicating that they were influenced by both a depth and tree species related variation in soil chemistry, as well as understorey vegetation biomass (Table 1). Additionally, the ectomycorrhizal : saprotrophic fungal ratio was positively correlated with the SOC stock (Fig. S5).

**Fig. 5:**
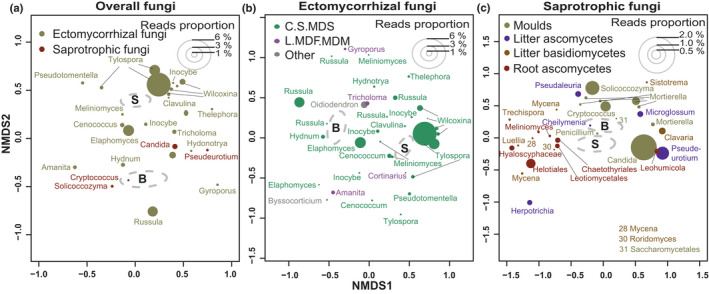
Global nonmetric multidimensional scaling (NMDS) ordination analysis for bacterial and fungal community composition. The species plots of (a) overall fungi, (b) ectomycorrhizal fungi and (c) saprotrophic fungi are based on total operational taxonomic units (OTUs) compositions (as in Fig. 2), but the most common OTUs (> 0.6% of total reads) are visualized. The size of the circles in each panel shows relative abundance of the OTUs and colour coded according to fungal guilds (a), ectomycorrhizal fungi exploration types (b) and saprotrophic fungi functionality (c). The dotted grey colour ellipses (95% confidence interval) represent centroid for birch (B) and Norway spruce (S). If the abundance optima of OTU (i.e. circle of OTU) in ordination space is closer to birch ellipse, it indicates their more affinity towards birch stands, whereas if abundance optima is closer to Norway spruce it shows contrasting patterns. The abbreviation C.S.MDS represent contact, short, medium distance smooth extrametrical mycelium morphology (EMM) exploration types and L.MDF.MDM denote long and medium distance fringe and mat EMM exploration types.

The relative abundance of Basidiomycota was significantly higher in spruce stands (58% reads) compared with birch stands (50% reads; Table S3). The order Atheliales was more abundant in spruce stands, whereas Russulales tended to be more abundant in birch stands (Table S3). The overall abundance of Ascomycota was, however, higher in birch (45% reads) relative to spruce (39% reads) stands, particularly for the orders Helotiales and Eurotiales, whereas the order Pezizales was more abundant in spruce stands (Table S3). In addition, the relative abundance of the Glomeromycotina, which was represented solely by the order Archaeosporales, was higher in the birch stands (Table S3). Ectomycorrhizal fungal OTUs with taxonomic affinities to *Russula*, *Elaphomyces* and *Gyroporous* showed an affinity with birch stands, whereas *Tylospora*, *Pseudotomentella* and *Wilcoxina* were more likely to occur in spruce stands (Fig. 5a). Ectomycorrhizal fungal genera with contact, short, medium distance smooth morphology (*Wilcoxina*, *Tylospora* and *Pseudotomentella*) occurred more frequently in spruce than in birch stands, whereas genera with long and medium distance, fringe and mat morphology (*Gyroporous*, *Amanita* and *Tricholoma*) were more frequent in birch than in spruce stands (Fig. 5b). Amongst the saprotrophs, OTUs with taxonomic affinities to the genera *Solicoccozyma* and *Cryptococcus* were more abundant in birch soil (Fig. 5a), whereas those with affinity to Helotiales and *Candida* were more abundant in spruce stands (Fig. 5c). When combining information on tree species and soil layer, mould and yeast genera such as *Candida*, *Cryptococcus*, *Mortierella* and *Solicoccozyma* were more abundant in birch mineral soil (species optima towards NMDS score > 1.0, representing birch mineral soil) (Fig. 5c). In contrast, the litter basidiomycetes *Mycena*, *Luellia* and *Trechispora* and root ascomycetes Helotiales and Hyaloscyphaceae were more abundant in the spruce LFH layer (species optima towards NMDS score > −0.5, representing birch mineral soil).

## Discussion

We found that a change in tree species from birch to spruce significantly impacted the composition of belowground microbial communities. In accordance with Hypothesis 1, the tree species effect was more pronounced for fungi compared with bacteria, and was reflected by higher fungal biomass in the spruce LFH layer. Our data also showed notable effects of tree species on both microbial groups in the LFH layer compared with the mineral soil, thus supporting Hypothesis 2. The effect was also associated with a decline in microbial richness, related to a change in understorey vegetation biomass (Hypothesis 3), and increases and reductions in the relative abundances of ectomycorrhizal fungi in the mineral soil and LFH layer of the spruce stands, respectively.

### Relationships of fungal guilds and biomass with SOC stocks

The overall fungal biomass did not vary between the spruce and birch stands. Nevertheless, the LFH layer in spruce stands contained significantly greater fungal biomass than LFH in birch stands, and showed a positive relationship with SOC stock. Whereas Lindahl *et al*. (2021) observed that the SOC stock in the organic topsoil was 33% lower in the presence of *Cortinarius acutus* s.l., we found no association between fungal taxa and SOC and N stocks. The abundance of *Cortinarius* spp., ectomycorrhizal fungi with strong litter decomposition activities (Bödeker *et al*., 2014), was low in the stands studied here, which was expected since *Cortinarius* spp. to a large extent dominate in older (> 100 yr) coniferous stands (Kyaschenko *et al*., 2017a). The limited presence of *Cortinarius*, which may potentially be due to competition with the abundant early successional ectomycorrhizal species belonging to the order Atheliales, such as *Tylospora* fungi (Wallander *et al*., 2010; Kyaschenko *et al*., 2017b; Lindahl *et al*., 2021), may hypothetically be an indirect driving factor for the accumulation of SOC in the LFH layer of the spruce stands. Further, the ectomycorrhizal : saprotrophic fungi ratio was positively correlated with SOC, suggesting a ‘Gadgil effect’ in the spruce stands related to a potential inhibition of fungal decomposition by ectomycorrhizal fungi through their competition with saprotrophs for organically bound N (Gadgil & Gadgil, 1971; Querejeta *et al*., 2021). However, the significantly larger SOC stock in the saprotroph‐dominated LFH layer of spruce stands suggests that the properties of incoming litter were a significant factor for the changes in SOC stocks and microbial community composition. This view is supported by the relative enrichment of ectomycorrhizal fungi in the deeper mineral soil, which contrasted with the pattern observed in birch stands for fungal guilds, as well as the absence of a relationship between fungal guilds and SOC and N stocks.

The higher SOC stock of the LFH layer of the spruce stands may be related to inputs of both aboveground and belowground litter (Kriiska *et al*., 2019). In the spruce stands, the root litter is to a large extent located in the LFH layer (Puhe, 2003), and the fine root production and biomass of spruce trees may potentially be higher relative to birch (Hansson *et al*., 2013b). Due to the significantly higher understorey vegetation biomass in birch relative to spruce stands (Kjønaas *et al*., 2021), the total flux of aboveground and belowground litter may be similar between the tree species, as found by Hansson *et al*. (2013a). However, the chemical composition of the litter is expected to differ between the stand types. The litter decomposition rate in the LFH layer of spruce stands relative to birch has been found to be significantly lower (Kjønaas *et al*., 2021), and significantly related to the more complex chemical nature and higher C : N ratio of SOM under spruce (M. Hansen, T. G. Bárcena, & O. J. Kjønaas, unpublished). This is also due to the almost absence of understorey vascular plant litter in the spruce stands. Hence, an increase in saprotroph colonization and mycelial growth in fresh needles and litter is expected, including a reallocation of assimilated N and mycelial growth from partially decomposed litter towards freshly fallen litter (Boberg *et al*., 2011). A reduced decomposition rate of partially decayed litter may conserve C in the existing organic matter pool. To conclude, more research on the causal relationships and the relative importance of various factors for SOC stocks is needed.

### Spruce plantation lowers soil microbial richness

The significant declines in bacterial and fungal richness in soil under spruce stands, with the largest changes being observed in the LFH layer, corroborate the observations of Danielsen *et al*. (2021), who also found that the replacement of birch with spruce in western Norway negatively affects fungal richness in the separate L and H layers. Tree species‐mediated changes in understorey vegetation biomass was the major predictor for the loss of richness for both microbial groups in the current study. The understorey vegetation of the spruce stands was dominated by bryophytes, with especially high cover and biomass of a few species of mosses at the locations with a northern aspect, whereas the understorey vegetation was very sparse at the location with a southern aspect (Kjønaas *et al*., 2021). Spruce litter and bryophytes generally have a high C : N ratio (Cools *et al*., 2014; Högberg *et al*., 2017), which can create unfavourable growth conditions for certain species of bacteria and fungi, with consequent effects on richness. In contrast, the understorey vegetation of the more open birch stands included a range of dwarf shrubs, graminoids, herbs, ferns and bryophytes (Kjønaas *et al*., 2021). Chemically and physically varied substrates create diverse niches and resource availabilities that facilitate soil chemical heterogeneity and enhance facilitative as well as competitive interactions (Chapman & Newman, 2010). This supports the higher bacterial (Lladó *et al*., 2017) and fungal (decomposers, root‐ and litter‐associated fungi) (Baldrian, 2017) richness observed here in soil under birch relative to that under spruce. The higher richness of Glomeromycota (Archaeosporales) in birch stands may be attributed to the higher vascular plant diversity, due to the symbioses formed by these fungi with specific plant hosts (Hoysted *et al*., 2019). An increase in soil microbial diversity has previously been found to be associated with vegetation changes from monocultures of *Betula pendula* or *Salix caprea* to two‐ or three‐species mixed stands of *Alnus* spp., *Larix decidua*, *Picea* spp., *Pinus* spp., *Quercus robur* and *Tilia cordata* (Šnajdr *et al*., 2013; Urbanová *et al*., 2015). Similarly, litter bag experiments have also shown a positive association between soil fungal richness and leaf and root litter diversity (Otsing *et al*., 2018).

### Spruce plantation alters fungal community composition

Spruce had clear effects on the composition of belowground fungal communities, with stronger effects in the LFH layer compared with the mineral soil. Compared with the more freely available particulate organic matter which dominates the forest floor, in mineral soil, the protection of organic matter by mineral particles reduces the availability of substrates for decomposers (Lavallee *et al*., 2020). The reduction in the availability of substrate with soil depth may explain the declining tree species effect on the fungal communities, as also observed in previous studies (Lindahl *et al*., 2007; Urbanová *et al*., 2015; Baldrian, 2017; Mundra *et al*., 2021).

Our results are in agreement with those of Danielsen *et al*. (2021), who also found shifts in fungal communities in the L and H layers following a change from birch to spruce forest. The variation in ectomycorrhizal fungal composition between stand types included significantly higher dominance of the basidiomycete *Tylospora* sp. and the ascomycete *Wilcoxina* sp. in spruce stands, and the basidiomycetes *Russula* spp. and *Gyroporus* spp. and the ascomycete *Elaphomyces* sp. in native birch stands. These differences concur with the previously reported dominance of *Russula* and *Elaphomyces* in beech and mixed forests and *Tylospora* in coniferous forests (Uroz *et al*., 2016; Asplund *et al*., 2018b), indicating the importance of host specificity. A higher abundance of the order Atheliales has been observed in *c*. 34‐yr‐old *Pinus*‐dominated stands mixed with *Picea*, with *Tylospora* being more frequent in younger *Picea* stands (12–34 yr) (Kyaschenko *et al*., 2017a). Species of *Russula*, which were more dominant in birch stands, tend to dominate in older stands (> 100 yr) (Kyaschenko *et al*., 2017a). They have a better nutrient acquisition strategy, and possess competitive advantages due to their efficient N‐ and phosphorus (P)‐mobilizing enzyme systems (Kyaschenko *et al*., 2017a). The increased abundance of the ectomycorrhizal fungal genera *Wilcoxina*, *Tylospora* and *Pseudotomentella* in spruce, and *Gyroporus*, *Amanita* and *Tricholoma* in birch stands, can also be linked with their nutrient foraging strategy based on their exploration type morphology (Agerer, 2001). Sterkenburg *et al*. (2015) reported the dominance of fungi with short‐ranging exploration type morphology (particularly *Tylospora* sp.) in fertile spruce‐dominated stands (Methods S1). Coniferous boreal forests generally favours the short‐distance, contact and smooth exploration types associated with fungi such as *Wilcoxina*, *Tylospora* and *Pseudotomentella* (Lilleskov *et al*., 2002; Sterkenburg *et al*., 2015). These taxa can access labile nutrients such as amino acids and ammonium. In contrast, fungi with more far‐reaching exploration types, such as *Gyroporus*, *Amanita* and *Tricholoma*, are seemingly better adapted to conditions in mineral soils of birch stands. We also found that species belonging to the orders Tremellales, Pezizales and Archaeorhizomycetales were more common in soil under birch compared with that under spruce. Some of these taxa act potentially as root‐associated mutualists (Menkis *et al*., 2014) and are associated with ericaceous plants such as bilberry, which were commonly found in the birch stands. Saprotrophic taxa such as *Candida*, *Solicoccozyma*, *Cryptococcus* and *Mortierella*, which were found to be common in the deeper mineral soil layers (M2–M3) of the birch stands, are generally favoured in nutrient‐rich and moist environments (Botha, 2011).

The saprotrophic genus *Mycena*, which had a higher relative abundance in the spruce LFH layer, is characterized by manganese (Mn)‐peroxidase activity, which is pivotal to the turnover of organic matter in spruce stands (Clemmensen *et al*., 2015; Kyaschenko *et al*., 2017b). Asplund *et al*. (2018a) showed in a study on *in situ* reciprocal litter incubation that the genus not only colonized spruce litter transplanted into beech forest, but accelerated the decomposition process due to its lignin decomposing ability. The higher abundance of *Mycena* in the spruce LFH layer concur with Asplund *et al*. (2018a, 2018b,2018a, 2018b), and suggests that it may have a crucial role in C cycling processes, particularly in the LFH layer of spruce stands.

### Spruce plantation alters bacterial communities

Bacteria are expected to respond rapidly to changes in their immediate surroundings (Urbanová *et al*., 2015). Accordingly, we found that the change from birch to spruce altered soil bacterial communities, but that the impact was much weaker than that on the fungi. The interaction between plant litter and local environmental conditions (i.e. temperature and moisture) determine bacterial community structure and colonization in boreal soil (Urbanová *et al*., 2015), its enzyme activities (López‐Mondéjar *et al*., 2016), and affect rates of decomposition and heterotrophic respiration (Tláskal *et al*., 2021). Thus, the large difference between the understorey vegetation species in the birch and spruce stands (Kjønaas *et al*., 2021) was expected to affect microscale heterogeneity and the bacterial community structure.

Proteobacteria, Firmicutes and Actinobacteria are common, functionally active litter decomposer taxa found in forest soils (López‐Mondéjar *et al*., 2016; Angst *et al*., 2019), with soil pH values controlling their abundance (Urbanová *et al*., 2015). The dominance of Actinobacteria was higher in birch stands, where the soil in the LFH layer was slightly less acidic compared with the same layer under spruce (Kjønaas *et al*., 2021). The abundance of the phylum was also positively correlated with the SOC and N stocks in the M1 layer. Actinobacteria are efficient producers of extracellular hydrolytic enzymes (Větrovský *et al*., 2014), with their hydrolytic enzyme diversity and activity being more efficient in acidic soils (Lladó *et al*., 2016). The decomposition activity of these bacteria is hence likely to be more efficient in spruce relative to birch stands. They preferentially utilize the easily accessible C (Eilers *et al*., 2010) and further incorporate relatively more cellulose‐derived C than that incorporated by fungi (Berlemont & Martiny, 2015; López‐Mondéjar *et al*., 2016). Firmicutes tended to be more abundant in the spruce stands, and, as for the Actinobacteria, displayed a positive relationship with SOC and N stocks in the M1 layer. Firmicutes, with their ligninolytic activity, are crucial in lignin decomposition (Kellner *et al*., 2008). Furthermore, their necromass, with diverse macromolecular chemistry and recalcitrancy, produces SOC with longer retention times (Throckmorton *et al*., 2012), and may thereby assist in the build‐up of SOC in the spruce LFH layer.

### Conclusions

Our results show that a change from birch to spruce, as encouraged in Norway in recent years to increase long‐term soil C storage, affect the composition of microbial communities (more pronounced for fungi), increase the proportion of ectomycorrhizal fungi (more evident in the mineral soil), and diminishes soil bacterial and fungal richness. The accumulation of SOC within the LFH layer of spruce stands may be mediated by fungal biomass, as well as by shifts caused by tree species‐specific changes in understorey vegetation, litter characteristics and soil properties. A subsequent reduction of decomposition in the spruce stands and potential competitions as well as interactions between ectomycorrhizal fungi and saprotrophic fungal guilds, may enhance the C accumulation in the LFH layer. Observed changes in the relative abundance and microbial richness in the mineral soil were not reflected by changes in the SOC stock. The interactions and feedbacks in the forest floor of spruce stands may potentially result in increasing SOC stocks, but the extent and long‐term effects of this process are highly uncertain.

## Author contributions

SM, HK and OJK designed the research, OJK and HK secured the funding, OJK was responsible for sampling and data analysis on soils, and TØ and J‐FN conducted the fieldwork and data analysis on understorey vegetation. YR performed the fungal biomass analyses. SM did the DNA‐based laboratory work, performed the bioinformatic and statistical analyses and drafted the manuscript, while all co‐authors edited and commented on the manuscript.

## Supporting information


**Fig. S1** A map showing the four locations with stands of native birch and planted Norway spruce in western Norway.
**Fig. S2** Nonmetric multidimensional scaling ordination analysis for six replicated sample pairs.
**Fig. S3** Principal component analysis of understorey vegetation biomass and soil chemical properties.
**Fig. S4** Pure and shared effects of tree species (birch vs spruce), soil depth and other principal component analysis axes.
**Fig. S5** Relationships between ectomycorrhizal : saprotrophic fungi ratio and soil organic carbon (Mg C ha^−1^) stock of birch and Norway spruce stands.
**Methods S1** Detailed site descriptions, soil biotic and abiotic parameters.
**Notes S1** Overall sequence data characteristics and microbial community composition.
**Table S1** Linear mixed effect (LME) models analysing effects of biotic and abiotic factors on stock carbon (C) and nitrogen (N) from different soil layers.
**Table S2** Taxonomic distribution of the bacterial and fungal community compositional occurrences (richness).
**Table S3** Taxonomic distribution of the bacterial and fungal community compositional reads (abundances).
Please note: Wiley Blackwell are not responsible for the content or functionality of any Supporting Information supplied by the authors. Any queries (other than missing material) should be directed to the *New Phytologist* Central Office.

## Data Availability

The raw sequence datasets for bacteria and fungi along with mapping files are archived at Zenodo (https://zenodo.org), with a common doi: 10.5281/zenodo.4415050. The datasets are freely accessible.
