# Supplementary material for: Shift in tree species changes the belowground biota of boreal forests
Source: New Phytol. 2022 Apr 10;234(6):2073–87. doi: 10.1111/nph.18109 (PMC9325058; doi:10.1111/nph.18109)
Supplement: Supplementary file 1 — Fig. S1 A map showing the four locations with stands of native birch and planted Norway spruce in western Norway. Fig. S2 Nonmetric multidimensional scaling ordination analysis for six replicated sample pairs. Fig. S3 Principal component analysis of understorey vegetation biomass and soil chemical properties. Fig. S4 Pure and shared effects of tree species (birch vs spruce), soil depth and other principal component analysis axes. Fig. S5 Relationships between ectomycorrhizal : saprotrophic fungi ratio and soil organic carbon (Mg C ha−1) stock of birch and Norway spruce stands. Methods S1 Detailed site descriptions, soil biotic and abiotic parameters. Notes S1 Overall sequence data characteristics and microbial community composition. Table S1 Linear mixed effect (LME) models analysing effects of biotic and abiotic factors on stock carbon (C) and nitrogen (N) from different soil layers. Table S2 Taxonomic distribution of the bacterial and fungal community compositional occurrences (richness). Table S3 Taxonomic distribution of the bacterial and fungal community compositional reads (abundances). Please note: Wiley Blackwell are not responsible for the content or functionality of any Supporting Information supplied by the authors. Any queries (other than missing material) should be directed to the New Phytologist Central Office. [file NPH-234-2073-s001.pdf]

New Phytologist

New Phytologist Supporting Information

Article title: ***Shift in tree species changes the belowground biota of boreal forests***

Authors: Sunil Mundra, Håvard Kauserud, Tonje Økland, Jørn-Frode Nordbakken, Yngvild Ransedokken, O. Janne Kjønaas

Article acceptance date: 14 March 2022

The following Supporting Information is available for this article:

Methods S1

Notes, S1

Figs S1-S5

Tables S1-S3

Methods S1 ***Site description, experimental setup and sampling:*** The locations are positioned in the middle boreal vegetation zone. The locations Jølster I (61°30'39'' N, 6°17'54''E; 225-250 m amsl) and Jølster II (61°30'22''N, 6°12'46''E; 335-345 m amsl) are located on a relatively steep N-facing slopes; Ørsta (62°9'1.8'' N, 6°12'2'' E; 210 m amsl) is located on a relatively gentle E-NE facing slope, whereas Stranda (62°16'23'' N, 6°51'5'' E; 430 m amsl) on S-SE facing slopes (Kjønaas *et al.*, 2021). The height adjusted mean annual temperature (MAT) for Jølster I, Jølster II, Ørsta, and Stranda was 4.99 °C, 3.95 °C, 6.17 °C and 3.41 °C, respectively, based on modelled estimates of temperature on a 1x1 km grid (Kjønaas *et al.*, 2021), whereas mean annual precipitation (MAP) was 2394, 2614, 1951, and 1584, respectively, measured at the nearest meteorological station (period: 1961–90; source: www.eklima.no). The soil type is regosoil/podsol with a similar soil texture (sandy loam/loamy sand /silt loam) (Kjønaas *et al.*, 2021). The stands were fertile, with a site index (H_{40}) for spruce of 23, which corresponds to the average height at age 40 years (Kjønaas *et al.*, 2021).

References

Kjønaas OJ, Bárcena TG, Hylen G, Nordbakken J-F, Økland T. 2021. Boreal tree species change as a climate mitigation strategy: impact on ecosystem C and N stocks and soil nutrient levels. Ecosphere 2(11):e03826.10.1002/ecs2.3826.

Notes S1 ***Overall microbial composition:*** Average bacterial and fungal reads per samples were 22,332 (range: 6154-63,165) and 108,496 (range: 50,938-164,855); and per OTUs were 809 (range: 2:120,298) and 2539 (range: 1:784,064), respectively. Of total 29 detected bacterial phyla, Proteobacteria (31% reads; 31% OTUs) was the most common followed by Acidobacteria (25% reads; 17% OTUs), Firmicutes (17% reads; 6% OTUs), Actinobacteria (8% reads; 7% OTUs) and Planctomycetes (6% reads; 13% OTUs). At genera level, bacteria belonging to *Gp2*, *Enterococcus* and Gp1 were the most abundant (all >4% of total reads). Among 6 identified

fungal phyla, Basidiomycota (52% reads; 31% OTUs) were the most abundant whereas Ascomycota were OTU rich (44% reads; 60% OTUs), followed by Mucoromycota (3% reads; 4% OTUs). Rozellamycota, Glomeromycotina and Chytridiomycota contained < 1% of reads. *Tylospora*, *Russula*, *Wilcoxina* and *Elaphomyces* were the most abundant genera (all >4% of total reads). The 10 most abundant bacterial and fungal OTUs represented 23% and 26% of total reads, respectively. Samples that were sequenced twice showed strong correspondences for both bacterial (Mantel r = 0.728; P = 0.029; Fig S2a) and fungal (Mantel r = 0.963; P = 0.002; Fig S2b) communities, suggesting stronger inter-sample variation and captured similar communities when samples were sequenced twice.

Fig. S1 A map showing the four locations with stands of native birch and planted Norway spruce in western Norway. Within each location, three 144 m^2 plots were selected for sampling.

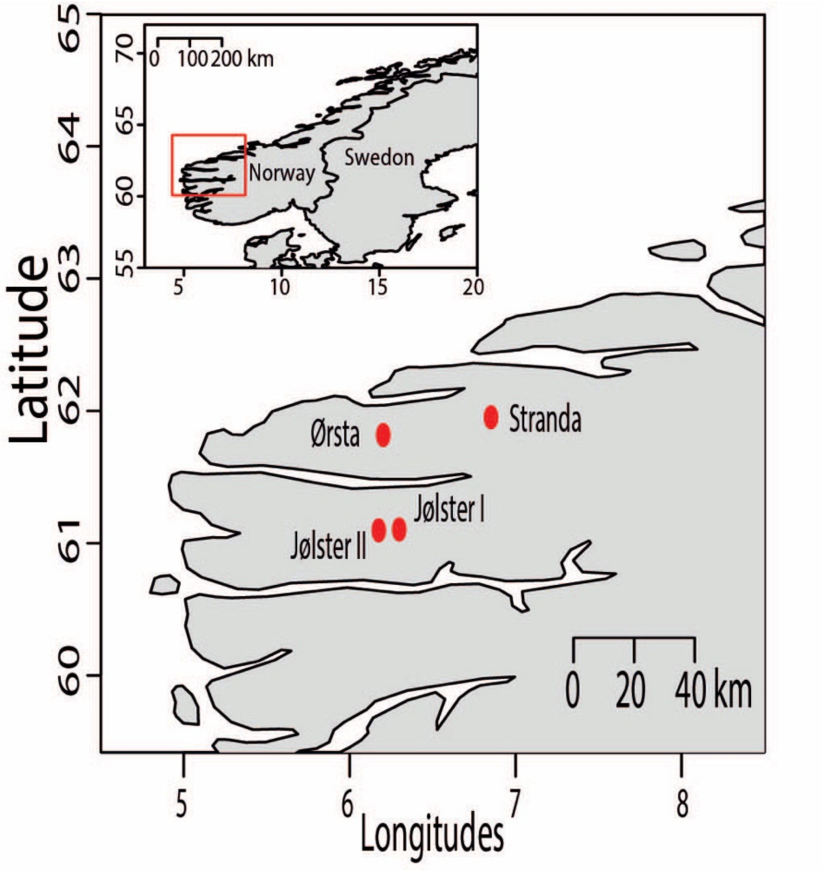

Fig. S2 Nonmetric multidimensional scaling (NMDS) ordination analysis for six replicated sample pairs. Samples that were sequenced twice, for (a) Bacteria and (b) fungi, during Miseq sequencing run, showing strong correspondence between pairs (also confirmed with Mantel test). Each replicate pair is connected by a line and labeled with the respective pair number.

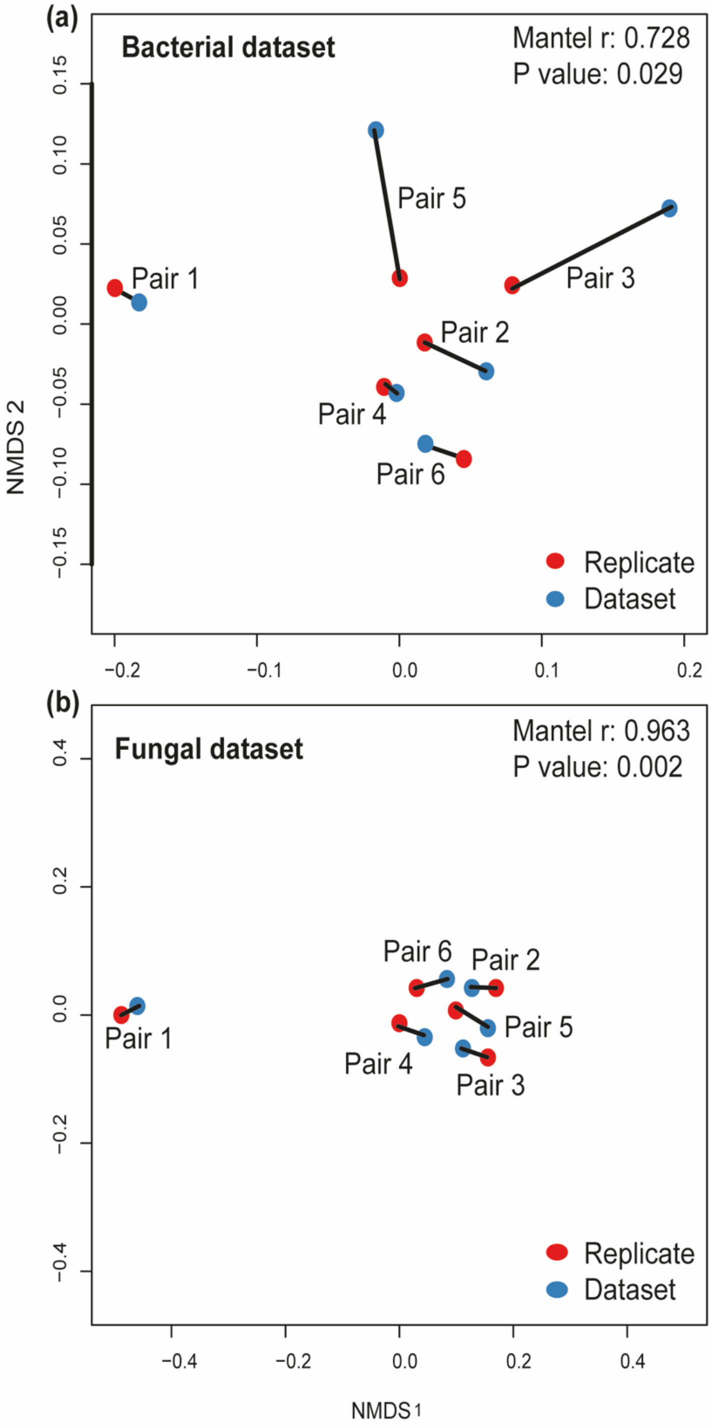

Fig. S3 Principal component analysis (PCA) of (a) understorey vegetation biomass and (b,c) soil chemical properties. The first PCA axis (PC1) score of plot (a) reflects aboveground tree species (birch vs Norway spruce) related variation in understorey vegetation biomass and is termed as "*understorey vegetation biomass index*". The negative PCA score indicates higher biomass in birch stands and positive score represent lower biomass in spruce The second PCA axis (soil PC2) score of plot (b) shows tree species related changes in soil chemistry and termed as "*tree species related soil variability index*". The first PCA axis (soil PC1) score plot (c) indicates depth related variation in soil chemistry and termed as "*depth related soil variability index*". The depth gradient include the forest floor (LFH) and three mineral soil layers: 0-5 cm (M1), 5-15 cm (M2), 15-30 cm (M3)). Soil chemistry include carbon (C%), nitrogen (N%), C/N ratio, soil pH and available exchangeable elements (exchangeable acidity (H), Aluminum (Al), Barium (Ba), calcium (Ca), Cadmium (Cd), Cobalt (Co), Chromium (Cr), Iron (Fe), Potassium (K); Magnesium (Mg), Manganese (Mn), Molybdenum (Mo), Sodium (Na), Nickel (Ni), Phosphorous (P), Lead (Pb), Sulphur (S), Silicon (Si), Strontium (Sr), Titanium (Ti) and Zinc (Zn)).

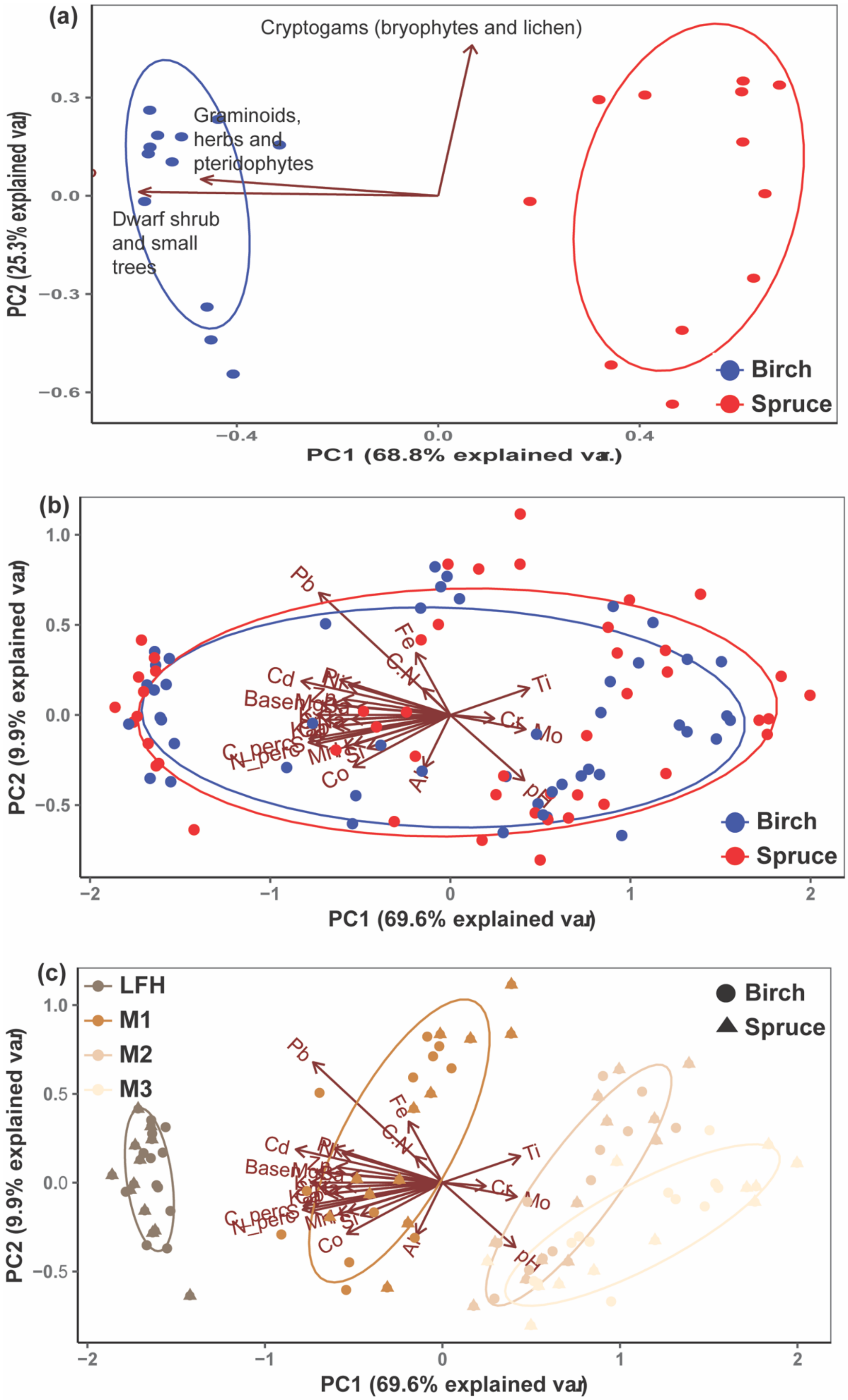
(a)
Cryptogams (bryophytes and lichen)
Graminoids, herbs and pteridophytes
Dwarf shrub and small trees
Birch
Spruce
PC2 (25.3% explained var.)
PC1 (68.8% explained var.)
(b)
Birch
Spruce
PC2 (9.9% explained var.)
PC1 (69.6% explained var.)
(c)
LFH
M1
M2
M3
Birch
Spruce
PC2 (9.9% explained var.)
PC1 (69.6% explained var.)

Fig. S4 Pure and shared effects of tree species (birch vs Norway spruce), soil depth (forest floor (LFH) and three mineral soil layers: 0-5 cm (M1), 5-15 cm (M2), and 15-30 cm (M3)), *depth related soil variability index* (soil PC1) and *understorey vegetation biomass index* (biomass PC1) on (a) bacterial and (b) and fungal communities, as derived from variation partitioning analysis. Different colour of venn diagram represent different variation explained by different variable (tree species, soil depth, soil PC1 and biomass PC1). Overlaps in venn diagram shown with orange colour indicates shared effect of soil depth and dark blue colour represent shared effect of tree species and soil PC1. Statistics denote the proportion of explained variation and values <0 are not shown.

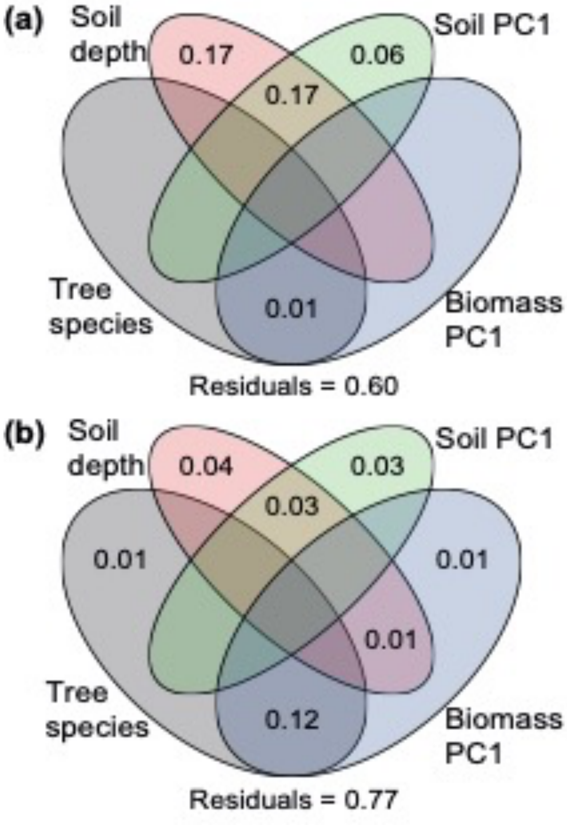

Fig. S5 Relationships between ectomycorrhizal:saprotrophic fungi ratio and soil organic carbon (SOC; Mg C ha^{-1}) stock down to 30 cm of birch and Norway spruce stands. The line represents a fitted linear regression. The SOC data was transformed to zero skewness i.e. normal distribution and scaled on a 0–1 level prior to plotting.

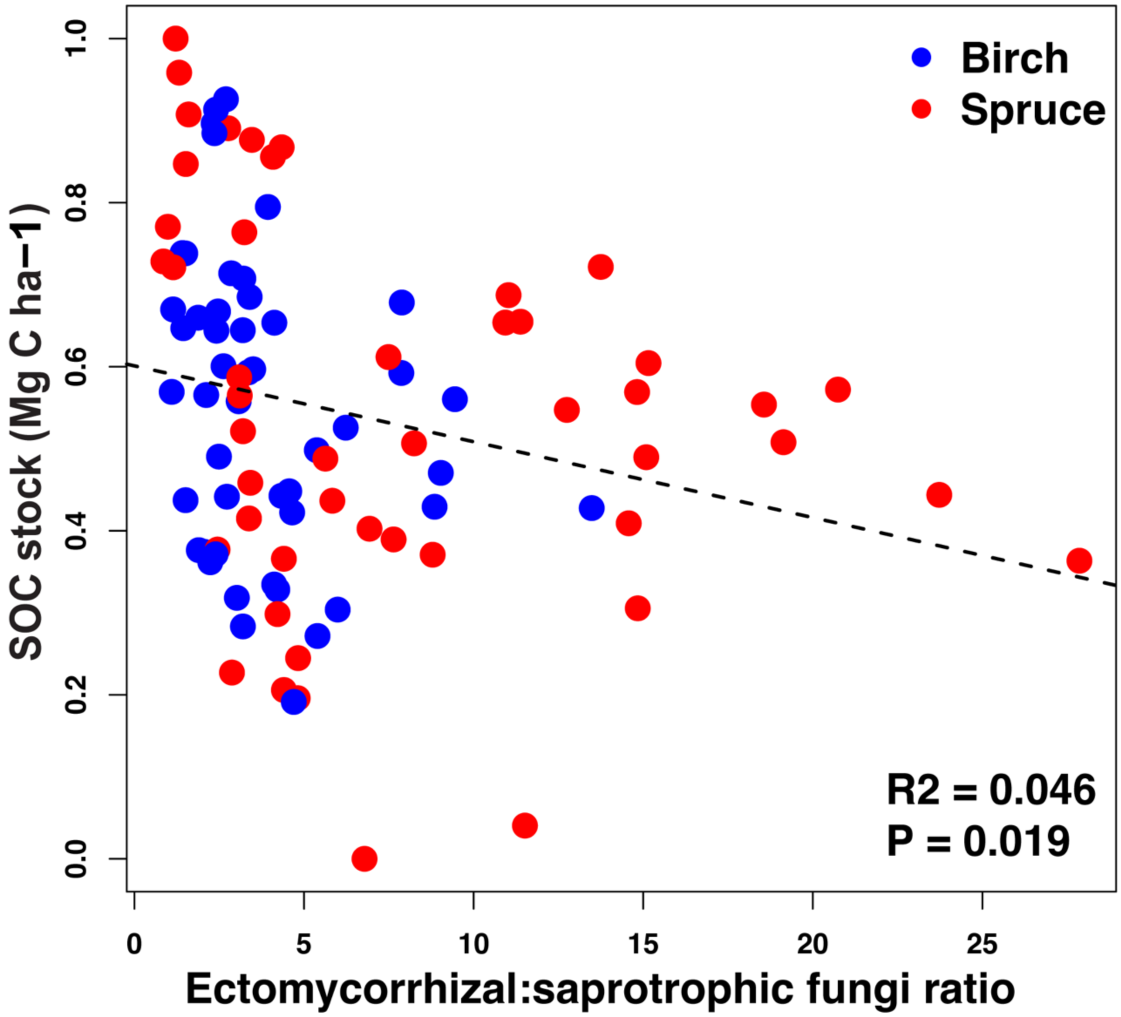

Table S1 Linear mixed effect (lme) models analyzing effects of biotic and abiotic factors on soil organic carbon (SOC Mg C ha-1) and nitrogen (N) from different soil layers. Response of soil C and N stock was examined against fixed effect variables: amount of ergosterol, bacterial and fungal richness, relative abundance of fungal functional guilds, and proportions of dominating bacterial phyla Numbers in bold represent significant ($p < 0.05$).

	LFH layer				M1 layer				M2 layer				M3 layer			
	SOC stock		N stock		SOC stock		N stock		SOC stock		N stock		SOC stock		N stock	
Fungi	t-value	p-value	t-value	p-value	t-value	p-value	t-value	p-value	t-value	p-value	t-value	p-value	t-value	p-value	t-value	p-value
Ectomycorrhizal fungi	0.93	0.363	0.35	0.732	-0.23	0.821	-0.68	0.504	-0.62	0.540	-1.51	0.148	-1.36	0.188	-1.11	0.281
Saprotrophic fungi	1.60	0.126	-0.41	0.683	0.70	0.490	1.23	0.233	-0.24	0.812	0.59	0.560	0.97	0.346	0.74	0.471
Ectomycorrhizal: saprotrophic fungi ratio	-0.41	0.687	0.55	0.589	-0.74	0.467	-0.80	0.433	-0.56	0.580	-1.14	0.269	0.15	0.884	0.14	0.891
Bacteria																
Proteobacteria	-1.94	0.068	-0.39	0.702	-2.10	**0.049**	-2.13	**0.047**	-1.05	0.307	-0.97	0.344	-1.04	0.312	-0.77	0.452
Acidobacteria	1.22	0.239	-0.55	0.592	-2.43	**0.025**	-1.97	0.064	0.99	0.335	0.73	0.475	1.21	0.242	0.59	0.560
Firmicutes	1.28	0.215	0.87	0.393	2.35	**0.030**	2.19	**0.041**	-1.00	0.330	-0.50	0.622	-0.64	0.530	-0.40	0.694
Actinobacteria	0.10	0.919	0.63	0.533	-2.22	**0.039**	-2.26	**0.036**	-1.04	0.311	-0.26	0.798	-1.03	0.318	0.09	0.930
Planctomycetes	-0.02	0.981	1.05	0.306	-0.90	0.382	-1.06	0.304	1.71	0.103	-0.08	0.934	0.20	0.846	-0.39	0.699
Verrucomicrobia	-0.10	0.919	-0.34	0.739	0.35	0.729	-0.16	0.873	2.07	0.052	-0.07	0.947	0.07	0.947	-0.56	0.583
Chloroflexi	1.65	0.115	2.26	**0.036**	-0.30	0.768	-0.03	0.978	1.09	0.288	1.41	0.173	1.82	0.085	1.85	0.079
Bacteroidetes	-1.64	0.118	-2.10	**0.049**	-1.15	0.266	-1.43	0.168	0.25	0.808	-0.16	0.876	-0.18	0.858	-0.39	0.704
Ergosterol	2.34	**0.030**	0.07	0.948	0.99	0.333	0.44	0.667	2.29	0.033	1.37	0.188	2.05	0.054	1.70	0.105

Table S2 Taxonomic (phylum and order level) distribution of the bacterial and fungal community compositional occurrences (richness) in the full dataset (total), subset of tree species (overall; birch vs Norway spruce) and soil depth (litter and humus (LFH), mineral soil: 0-5 cm (M1), 5-15 cm (M2), 15-30 cm (M3)). Order with >1 % of total reads are shown here. *gis = Order incertae sedis #ois = Order incertae sedis.

Taxonomy	Total	Overall		LFH		M1		M2		M3	
		Birch	Spruce	Birch	Spruce	Birch	Spruce	Birch	Spruce	Birch	Spruce
**Bacteria**											
Proteobacteria	31.4	31.5	31.2	33.8	33.7	31.5	31.0	30.5	30.3	30.2	29.9
Rhizobiales	5.5	5.4	5.6	5.4	5.7	5.3	5.7	5.3	5.4	5.7	5.7
Xanthomonadales	2.0	2.0	2.0	2.5	2.5	2.1	2.1	1.8	1.9	1.6	1.6
Rhodospirillales	6.4	6.3	6.4	6.7	6.9	6.6	6.7	6.2	6.3	5.8	5.7
Myxococcales	5.1	5.2	5.0	5.5	5.2	5.0	4.6	5.1	5.0	5.3	5.4
Burkholderiales	1.4	1.4	1.4	1.5	1.6	1.4	1.2	1.4	1.3	1.5	1.4
Rhodocyclales	1.0	1.0	1.0	1.0	1.0	0.9	0.8	1.0	1.0	1.0	1.0
Acidobacteria	17.2	17.0	17.4	16.3	17.1	17.0	17.9	17.1	17.0	17.8	17.6
Gp2	4.9	4.9	5.0	4.3	4.6	4.7	5.1	5.0	5.0	5.5	5.3
Gp1	2.5	2.5	2.6	2.4	2.5	2.6	2.7	2.5	2.5	2.6	2.6
Gp3	3.6	3.5	3.6	3.5	3.7	3.5	3.8	3.4	3.5	3.5	3.6
Gp6	0.7	0.8	0.7	0.6	0.6	0.8	0.7	0.8	0.8	0.8	0.8
Firmicutes	5.7	5.6	5.8	4.3	4.5	5.1	5.5	6.1	6.4	7.0	7.0
Lactobacillales	0.9	0.9	1.0	0.7	0.8	0.9	0.9	0.9	1.0	1.1	1.1
Bacillales	2.1	2.0	2.1	1.5	1.5	1.8	2.0	2.3	2.4	2.7	2.7
Clostridiales	1.5	1.5	1.5	1.3	1.1	1.4	1.4	1.6	1.7	1.8	1.7
Actinobacteria	7.2	7.3	7.1	8.2	7.9	7.5	6.7	6.9	6.7	6.8	6.9
Actinomycetales	3.3	3.3	3.2	4.0	3.9	3.4	3.1	3.0	2.9	2.9	2.9
Solirubrobacterales	1.3	1.3	1.2	1.5	1.5	1.3	1.2	1.1	1.1	1.1	1.1
Acidimicrobiales	2.0	2.0	1.9	1.8	1.8	2.0	1.8	2.0	2.1	2.1	2.1
Planctomycetes	12.5	12.4	12.7	11.7	11.5	13.0	13.3	12.7	13.0	12.2	12.8

Planctomycetales	12.3	12.1	12.4	11.3	11.2	12.7	13.1	12.4	12.8	12.0	12.6
Verrucomicrobia	4.9	4.9	5.0	5.5	5.3	5.1	5.2	4.8	4.9	4.3	4.3
Spartobacteria_gis*	1.4	1.4	1.4	1.4	1.4	1.4	1.4	1.3	1.3	1.3	1.3
Subdivision3_gis*	1.9	1.9	1.8	2.0	1.9	1.9	2.0	1.9	1.8	1.7	1.7
Chloroflexi	5.6	5.5	5.7	3.6	3.7	4.5	4.8	6.5	6.7	7.6	7.6
Ktedonobacterales	3.1	3.0	3.2	1.9	2.0	2.4	2.7	3.6	3.7	4.2	4.2
Bacteroidetes	5.1	5.3	4.8	6.8	6.3	5.6	4.8	4.7	4.4	4.1	3.7
Sphingobacteriales	3.4	3.6	3.3	4.6	4.4	3.6	3.3	3.1	3.0	2.8	2.5
Others	10.4	10.4	10.4	9.8	10.0	10.7	10.8	10.9	10.6	10.1	10.3
**Fungi**											
Basidiomycota	30.9	29.4	32.5	28.1	30.8	28.9	32.8	30.4	32.9	31.2	33.9
Agaricales	13.0	12.4	13.7	11.9	13.2	12.0	14.1	13.0	13.6	13.2	14.0
Atheliales	2.0	1.4	2.8	1.4	2.5	1.3	3.0	1.4	2.8	1.4	2.9
Russulales	3.2	3.2	3.3	2.7	2.8	3.2	3.3	3.4	3.4	3.9	3.8
Thelephorales	2.2	2.0	2.4	1.9	2.1	2.0	2.3	2.3	2.6	2.1	2.5
Cantharellales	1.6	1.6	1.6	1.4	1.5	1.5	1.6	1.7	1.7	1.8	1.8
Sebacinales	1.8	1.9	1.7	1.6	1.3	1.8	1.6	2.3	2.0	2.3	2.1
Boletales	0.7	0.7	0.7	0.6	0.4	0.8	0.7	0.8	0.8	0.9	0.8
Ascomycota	59.6	60.2	58.9	62.1	61.1	60.1	58.3	58.4	58.0	59.1	57.6
Helotiales	21.3	21.4	21.1	21.5	20.8	21.8	21.6	21.1	21.2	21.2	21.0
Leotiomycetes_ois#	6.6	6.3	6.9	5.2	6.4	6.3	7.9	7.0	7.1	7.3	6.4
Pezizales	1.6	1.4	1.8	1.2	1.3	1.4	1.8	1.6	2.0	1.7	2.1
Eurotiales	1.5	1.5	1.5	1.4	1.4	1.6	1.6	1.5	1.6	1.5	1.5
Chaetothyriales	6.2	6.3	6.2	7.4	8.2	6.4	5.7	5.6	5.0	5.1	5.1
Hysteriales	0.7	0.7	0.8	0.6	0.8	0.8	0.8	0.8	0.8	0.7	0.7
Saccharomycetales	0.5	0.5	0.5	0.3	0.3	0.5	0.6	0.6	0.7	0.7	0.7
Pezizomycotina_ois#	2.1	2.0	2.1	2.4	2.5	1.9	1.8	1.7	1.9	1.9	2.1
Unclassified Leotiomycetes	3.8	4.0	3.6	4.1	3.5	3.9	3.5	4.0	3.7	4.2	3.8

Venturiales	1.8	1.8	1.8	2.5	2.3	1.7	1.6	1.3	1.4	1.3	1.6
Mucoromycota	3.6	3.6	3.5	2.9	2.9	3.8	3.7	4.2	4.0	3.8	3.7
Mortierellales	2.4	2.5	2.3	2.0	1.8	2.6	2.3	2.9	2.6	2.5	2.4
Rozellomycota	2.0	2.2	1.7	2.2	1.2	2.5	2.0	2.3	2.0	1.6	1.7
Glomeromycotina	0.5	0.8	0.2	0.5	0.2	0.7	0.1	1.2	0.4	1.2	0.2
Chytridiomycota	0.1	0.2	0.1	0.2	0.2	0.2	0.1	0.1	0.1	0.1	0.1
Unclassified Fungi	3.4	3.6	3.1	3.9	3.6	3.8	3.0	3.4	2.7	3.1	2.8

Table S3 Taxonomic (phylum and order level) distribution of the bacterial and fungal community compositional reads (abundances) in the full dataset (total), in subsets of tree species (overall; average in each of birch and Norway spruce) and with soil depth (litter and humus (LFH), mineral soil 0-5 cm (M1), 5-15 cm (M2), 15-30 cm (M3)). Order with >1 % of total reads are shown here. *gis = Order incertae sedis #ois = Order incertae sedis.

Taxonomy	Total	Overall		LFH		M1		M2		M3	
		Birch	Spruce	Birch	Spruce	Birch	Spruce	Birch	Spruce	Birch	Spruce
**Bacteria**											
Proteobacteria	31.9	32.5	31.4	40.5	37.5	32.3	32.8	31.4	30.6	26.4	26.2
Rhizobiales	13.5	13.7	13.3	14.3	13.7	13.9	13.9	15.2	14.2	11.6	11.6
Xanthomonadales	4.3	3.9	4.6	5.1	5.8	3.8	5.4	3.5	4.2	3.3	3.6
Rhodospirillales	4.2	4.2	4.2	5.5	4.6	4.6	4.9	3.8	3.9	3.2	3.5
Myxococcales	2.0	2.2	1.8	3.3	2.3	2.3	2.0	1.8	1.5	1.7	1.5
Burkholderiales	1.6	1.8	1.5	3.2	3.5	1.3	0.8	1.3	0.9	1.3	0.8
Rhodocyclales	1.3	1.3	1.4	1.0	0.8	1.2	1.2	1.4	1.6	1.6	1.8
Acidobacteria	24.1	23.6	24.5	17.4	19.6	20.9	23.4	26.6	26.5	28.9	27.6
Gp2	10.8	10.2	11.3	5.2	6.6	7.7	10.3	12.4	12.6	15.3	14.6
Gp1	4.0	4.0	4.0	2.8	3.1	4.3	4.8	4.9	4.8	4.1	3.5
Gp3	3.0	3.0	3.0	3.5	3.1	3.2	3.2	3.1	3.1	2.5	2.8
Gp6	1.5	1.6	1.5	0.7	0.5	1.2	1.0	2.1	2.1	2.1	2.0
Firmicutes	14.8	13.8	15.6	4.4	4.9	15.2	13.8	14.7	17.9	20.3	23.5
Lactobacillales	6.8	6.1	7.3	2.2	2.6	8.9	7.7	5.9	8.5	7.1	9.9
Bacillales	6.7	6.4	6.9	1.6	1.7	5.6	5.5	7.4	8.0	10.5	11.0
Clostridiales	1.0	1.0	1.1	0.4	0.4	0.4	0.4	1.0	1.0	2.1	2.1
Actinobacteria	7.9	8.4	7.5	12.0	11.0	9.4	8.0	7.0	6.2	5.7	5.4
Actinomycetales	4.2	4.5	4.0	7.4	6.9	5.6	5.2	3.2	2.7	2.1	2.0
Solirubrobacterales	1.9	2.0	1.9	2.7	2.6	2.2	1.7	1.8	1.7	1.4	1.5
Acidimicrobiales	1.3	1.3	1.2	1.2	1.1	1.0	0.9	1.4	1.3	1.7	1.6
Planctomycetes	6.6	6.9	6.3	7.4	7.1	8.9	8.3	6.9	6.1	4.5	4.5

Planctomycetales	6.5	6.9	6.3	7.3	7.0	8.8	8.3	6.9	6.1	4.4	4.5
Verrucomicrobia	4.0	3.9	4.1	5.3	5.7	4.6	5.2	3.7	3.6	2.2	2.3
Spartobacteria_gis*	2.0	1.9	2.1	1.5	1.5	2.4	2.8	2.4	2.4	1.4	1.7
Subdivision3_gis*	1.0	1.1	1.0	1.9	1.8	1.3	1.2	0.8	0.7	0.5	0.4
Chloroflexi	3.7	3.7	3.6	1.2	1.2	1.7	1.8	4.4	4.3	7.3	6.3
Ktedonobacterales	2.3	2.3	2.4	0.6	0.7	1.1	1.2	2.7	2.8	4.8	4.1
Bacteroidetes	3.3	3.5	3.2	7.8	8.2	3.5	2.6	1.9	1.6	1.1	0.8
Sphingobacteriales	2.7	2.8	2.6	6.3	6.9	2.9	2.2	1.5	1.3	0.7	0.6
Others	3.7	3.6	3.8	4.0	4.8	3.5	3.9	3.4	3.2	3.6	3.3
**Fungi**											
Basidiomycota	54.0	49.8	57.7	44.3	50.6	49.2	62.8	54.2	59.2	50.6	57.8
Agaricales	15.8	16.4	15.4	17.3	14.7	18.0	24.6	17.4	14.0	12.9	9.6
Atheliales	13.7	3.3	23.2	6.2	15.5	4.3	19.1	2.0	26.8	1.3	29.2
Russulales	11.1	15.9	6.7	9.9	3.3	11.8	5.7	20.4	7.1	20.3	10.0
Thelephorales	3.5	2.5	4.4	2.4	6.9	2.2	6.9	1.7	2.9	3.9	1.6
Cantharellales	2.9	3.1	2.8	2.2	3.7	2.3	2.7	4.7	2.9	3.0	2.0
Sebacinales	2.0	2.3	1.7	1.6	0.5	2.8	1.3	2.6	2.5	2.2	2.2
Boletales	1.2	1.8	0.6	0.2	0.1	0.2	0.4	1.2	0.6	5.5	1.2
Ascomycota	41.7	44.9	38.8	51.5	46.3	44.9	33.8	40.3	37.3	44.0	38.4
Helotiales	11.1	12.6	9.8	15.6	12.5	15.0	9.5	10.5	9.0	9.9	8.5
Leotiomycetes_ois#	6.4	6.3	6.4	4.2	8.5	4.0	7.4	7.8	5.9	8.9	4.4
Pezizales	5.6	2.5	8.3	1.5	0.9	2.0	3.5	2.6	10.9	3.8	16.2
Eurotiales	4.0	5.3	2.9	7.0	3.6	7.4	4.8	5.2	2.9	1.8	0.6
Chaetothyriales	2.4	3.6	1.2	5.3	3.0	2.3	1.1	3.0	0.5	4.0	0.6
Hysteriales	2.3	1.7	2.8	1.9	5.4	3.1	3.1	1.1	2.1	0.8	0.9
Saccharomycetales	1.5	1.7	1.4	0.2	0.1	2.1	0.7	1.6	1.7	2.8	2.7
Pezizomycotina_ois#	1.4	1.8	1.1	1.1	1.4	0.4	0.2	1.9	1.0	3.5	1.7
Unclassified Leotiomycetes	1.4	1.8	0.9	2.4	0.8	1.6	1.2	1.6	1.2	1.8	0.6

Venturiales	1.3	1.5	1.1	4.6	4.0	1.5	0.4	0.2	0.2	0.2	0.3
Mucoromycota	2.2	2.1	2.3	1.0	0.9	2.3	2.2	2.8	2.8	2.4	3.1
Mortierellales	1.9	1.7	2.0	0.9	0.8	1.8	1.7	2.1	2.3	2.1	2.8
Rozellomycota	0.2	0.4	0.1	0.3	0.1	0.5	0.2	0.4	0.2	0.3	0.1
Glomeromycotina	0.0	0.1	0.0	0.0	0.0	0.1	0.0	0.1	0.0	0.2	0.0
Chytridiomycota	0.0	0.0	0.0	0.0	0.0	0.0	0.0	0.0	0.0	0.0	0.0
Unclassified Fungi	1.8	2.7	1.0	2.8	2.1	3.2	1.0	2.2	0.5	2.6	0.7
